# The effect of standard heat and filtration processing procedures on antimicrobial activity and hydrogen peroxide levels in honey

**DOI:** 10.3389/fmicb.2012.00265

**Published:** 2012-07-27

**Authors:** Cuilan Chen, Leona T. Campbell, Shona E. Blair, Dee A. Carter

**Affiliations:** ^1^School of Molecular Bioscience, University of Sydney, SydneyNSW, Australia; ^2^School of Biotechnology and Biomolecular Sciences, University of New South Wales, SydneyNSW, Australia

**Keywords:** honey, Australia, *Staphylococcus aureus*, *Candida albicans*, hydrogen peroxide, heat treatment

## Abstract

There is increasing interest in the antimicrobial properties of honey. In most honey types, antimicrobial activity is due to the generation of hydrogen peroxide (H_2_O_2_), but this can vary greatly among samples. Honey is a complex product and other components may modulate activity, which can be further affected by commercial processing procedures. In this study we examined honey derived from three native Australian floral sources that had previously been associated with H_2_O_2_-dependent activity. Antibacterial activity was seen in four red stringybark samples only, and ranged from 12 to 21.1% phenol equivalence against *Staphylococcus aureus*. Antifungal activity ranged from MIC values of 19–38.3% (w/v) against *Candida albicans*, and all samples were significantly more active than an osmotically equivalent sugar solution. All honey samples were provided unprocessed and following commercial processing. Processing was usually detrimental to antimicrobial activity, but occasionally the reverse was seen and activity increased. H_2_O_2_ levels varied from 0 to 1017 μM, and although samples with no H_2_O_2_ had little or no antimicrobial activity, some samples had relatively high H_2_O_2_ levels yet no antimicrobial activity. In samples where H_2_O_2_ was detected, the correlation with antibacterial activity was greater in the processed than in the unprocessed samples, suggesting other factors present in the honey influence this activity and are sensitive to heat treatment. Antifungal activity did not correlate with the level of H_2_O_2_ in honey samples, and overall it appeared that H_2_O_2_ alone was not sufficient to inhibit *C. albicans*. We conclude that floral source and H_2_O_2_ levels are not reliable predictors of the antimicrobial activity of honey, which currently can only be assessed by standardized antimicrobial testing. Heat processing should be reduced where possible, and honey destined for medicinal use should be retested post-processing to ensure that activity levels have not changed.

## Introduction

Honey has been widely used for thousands of years, not only in food and beverages but also for treating diseases (Blair and Carter, 2005). As a complex natural product, there are a variety of factors that contribute to the antimicrobial activity of honey. The primary antimicrobial component in most honeys is hydrogen peroxide (H_2_O_2_), which is produced by the bee-derived enzyme glucose oxidase (White et al., 1963). Certain honey types contain additional antimicrobial activity, which has been attributed to various different components including methylglyoxal (MGO), bee defensin-1, and other bee-derived compounds, florally derived phenolics, lysozyme, and other yet undetermined compounds (Estevinho et al., 2008; Irish et al., 2008; Mavric et al., 2008; Adams et al., 2009; Kwakman et al., 2010, 2011). Antimicrobial activity derived from these components has been grouped together and is generally referred to in the literature as “non-peroxide dependent” activity (Blair and Carter, 2005).

Honey is broad-spectrum and active against a range of different bacteria and fungi (Molan, 1992, 2009). Transcriptome and proteome studies on how bacteria respond to treatment have found honey to have a unique and multimodal mode of action (Blair et al., 2009; Packer et al., 2012). In addition, unlike most antibiotics, resistance to honey cannot be induced (Blair et al., 2009). These features make honey an attractive alternative treatment, particularly for topical application to skin and mucosal membranes (English et al., 2004; Chambers, 2006; Molan, 2006b).

Australia has a diverse, unique natural flora, and honey production is a multi-million dollar industry. Although predominantly destined for table use, some antimicrobial Australian honey is also produced and marketed. Recently, a survey of the antibacterial properties of honey derived from a wide range of Australian plants was undertaken that demonstrated the potential of Australian floral sources for the production of medical-grade honey (Irish et al., 2011). As well as finding H_2_O_2_- and MGO-type activity, this study found some honey samples had antimicrobial activity that was clearly different to known peroxide and non-peroxide activities.

The current study was undertaken to follow up some of the native Australian honeys investigated by Irish et al. (2011) in order to determine whether some of the more promising floral sources would produce consistently active honey, and to assess if new, as yet undefined non-peroxide activities could be identified. During the course of honey testing we were provided with samples that were completely unprocessed, and with aliquots of the same honey samples that undergone heating and filtration procedures used in the honey industry to remove wax and particulate matter and to prevent granulation. Heat treatment is relatively mild (~45°C for up to 8 h) and does not appear to affect MGO levels (Matheson and Murray, 2011) but might affect enzymes such as glucose oxidase or other non-peroxide factors. Since antimicrobial testing is generally conducted on raw, unprocessed honey, but heat and filtration are required to process honey, we were interested to see how antimicrobial activity was affected by these methods. Finally, as the addition of catalase reduced the antimicrobial activity in all samples to insignificant levels and there was no apparent non-peroxide activity, we assessed the levels of H_2_O_2_ to determine how this correlated with antimicrobial potency.

## Materials and methods

### Honey samples

All honeys were sourced and supplied by Beechworth Honey (Corowa, NSW, Australia) and are listed in Table 1. Honey derived from three native Australian floral sources were tested, with five independent samples of each. These included spotted gum (*Eucalyptus maculata*) (samples S1–S5), red stringybark (*Eucalyptus macrorrhyncha*) (samples R1–R5), and yellow box (*Eucalyptus melliodora*) (samples Y1–Y5). One mixed sample of canola/red stringybark (R6) and one pure sample of canola honey (*Brassica napus*) (C1) were also included. It should be noted that the identified plants only represent the major source of honey for that sample; honeys are rarely derived from only one species, and other floral sources may contribute to any one batch. An artificial honey (7.5 g sucrose, 37.5 g maltose, 167.5 g glucose, and 202.5 g fructose in 85 mL sterile water) was used to simulate osmotic factors due to the high sugar level in honey. Comvita UMF^®^ 18+ manuka honey (Te Puke, New Zealand) was used as a positive control in the phenol equivalence assay.

**Table 1:** **Antibacterial activity, antifungal activity, and hydrogen peroxide concentrations of honey samples before and after heat and filtering processes**.

		**Antibacterial activity (% phenol equivalence against *S. aureus*)**			**Antifungal activity (MIC % [w/v] honey against *C. albicans*)**			**Hydrogen peroxide concentration (μM)**		
**Honey ID**	**Honey type**	**Unprocessed**	**Processed**	**Change following processing^a^**	**Unprocessed^b^**	**Processed^b^**	**Change following processing^a^**	**Unprocessed**	**Processed**	**Change following processing (% remaining)^a^**
		**Mean ± SD**	**Mean ± SD**		**Mean ± SD**	**Mean ± SD**		**Mean ± SD**	**Mean ± SD**	
R1	Red stringybark	14.9 ± 0.9	11.9 ± 1.2	–3.0	28.3 ± 0.6^**^	18.0 ± 2.7^**^	–10.3^*^	526 ± 24.7	553 ± 31.8	26 (105)
R2	Red stringybark	21.2 ± 1.1	14.0 ± 0.4	–7.2^*^	19.0 ± 0.0^**^	20.0 ± 0.0^**^	1.0	796 ± 1.2	739 ± 20.0	–57 (93)
R3	Red stringybark	14.1 ± 0.9	11.4 ± 0.9	–2.7^*^	19.3 ± 0.6^**^	18.0 ± 1.4^*^	–1.3	1017 ± 10.6	627 ± 3.5	–390^*^ (62)
R4	Red stringybark	No activity	No activity	–	35.0 ± 0.0^**^	35.0 ± 0.0^**^	0.0	0	0	–
R5	Red stringybark	No activity	No activity	–	34.3 ± 0.6^**^	38.3 ± 2.1	4.0^*^	0	0	–
R6	Canola/Red stringybark	12.0 ± 1.6	No activity	–12.0^*^	24.3 ± 1.2^**^	28.5 ± 0.7^**^	4.2^*^	916 ± 1.8	321 ± 9.5	–595^*^ (35)
Y1	Yellowbox	No activity	No activity	–	35.0 ± 0.0^**^	37.3 ± 4.0	2.3	0	0	–
Y2	Yellowbox	No activity	No activity	–	38.0 ± 0.0^**^	39.0 ± 1.0	1.0	0	0	–
Y3	Yellowbox	No activity	No activity	–	29.0 ± 0.0^**^	27.5 ± 2.1	–1.5	543 ± 7.7	279 ± 9.4	–265^*^ (51)
Y4	Yellowbox	No activity	No activity	–	33.3 ± 1.2^**^	33.3 ± 1.2^**^	0.0	645 ± 7.1	0	–645^*^ (0)
Y5	Yellowbox	No activity	No activity	–	31.7 ± 0.6^**^	34.7 ± 0.6^**^	3.0^*^	488 ± 1.8	0	–488^*^ (0)
S1	Spotted gum	No activity	No activity	–	33.0 ± 1.0^**^	29.0 ± 2.9^**^	–4.0	151 ± 1.2	122 ± 7.1	–29 (81)
S2	Spotted gum	No activity	No activity	–	30.3 ± 0.6^**^	38.7 ± 2.9	8.4^*^	284 ± 7.6	0	–284^*^ (0)
S3	Spotted gum	No activity	No activity	–	35.7 ± 2.1^**^	40.7 ± 2.1	5.0^*^	0	0	–
S4	Spotted gum	No activity	No activity	–	36.3 ± 1.2^**^	38.0 ± 1.0^*^	1.7	0	0	–
S5	Spotted gum	No activity	No activity	–	30.3 ± 0.6^**^	36.3 ± 2.3	6.0^*^	426 ± 19.4	0	–426^*^ (0)
C1	Canola	No activity	No activity	–	38.3 ± 0.6^**^	42.5 ± 1.0^∞^	4.2^*^	754 ± 5.2	0	–754^*^ (0)
	Artificial honey	No activity	No activity		40.7 ± 2.9	40.7 ± 0.6	−	0	0	–

a Values in red indicate drop in activity or H_2_O_2_ concentration following processing; ^*^Denotes significant change p < 0.05.

b Significantly different MIC to artificial honey: Lower:

* p < 0.05;

** p < 0.01;

∞ p < 0.05.

### Honey treatment

“Unprocessed” and “processed” versions of each honey sample were supplied and tested. The unprocessed samples were supplied directly as they had been obtained from beekeepers and had not undergone any heating or filtration. Processed aliquots of each of the same honey samples had been subjected to standard commercial treatment at Beechworth Honey, which involved heating the bulk honey purchase to 45°C for 8 h and filtering with a 100 μm filter.

Immediately prior to antimicrobial tests, all honey samples were diluted and filtered through a 0.2 μm fliter (Millipore) in the laboratory to eliminate contaminating micro-organisms. For the assessment of non-peroxide activity, catalase (Sigma-Aldrich, USA) was added to samples at a final concentration of 2800 U/mL prior to testing.

### Assessment of antibacterial activity

The antibacterial activity of honey samples was assessed using the standard method described by Allen et al. (1991a). This measures the inhibition of *Staphylococcus aureus* strain ATCC 25923 (Oxoid, Hampshire, UK) by honey in an agar well diffusion assay and reports activity as equivalence to dilutions of phenol. Briefly, an 18 h culture of *S. aureus* grown in tryptone soy broth (TSB) was adjusted to 0.5 at A_540_ nm (approx. 5×10^7^ cells/ml). One hundred and fifty mL of molten, cooled nutrient agar (BD Difco, USA) was seeded with 100 μL of the prepared *S. aureus* culture and poured into a large square bioassay plate (245×245 mm; Corning). Plates were stored inverted at 4°C for use the next day, when wells were cut into the agar with a sterile 8 mm diameter cork borer. Each well was numbered, in duplicate, using a quasi-Latin square, which enabled duplicate samples to be placed randomly on the plate.

Fifty percent (w/v) of each honey sample, including the Comvita and the artificial honey, were prepared fresh for each assay in sterile deionized water, and incubated at 37°C with shaking at 200 rpm for 30 min to aid mixing. Diluted honey samples were then filter sterilized through 0.2 μm pore filters (Millipore) and mixed with equal volumes of either sterile deionized water for total activity testing, or freshly prepared 5600 U/mL catalase solution (Sigma-Aldrich, USA) for non-peroxide activity testing, to give a final concentration of 25% (w/v) honey. Aliquots of 100 μL of each solution were placed into wells of the assay plates.

Phenol (Sigma-Aldrich, USA) standards of 2, 3, 4, 5, 6, and 7% were prepared from a 10% w/v solution that was freshly made every four weeks in sterile deionized water and stored at 4°C. Aliquots of 100 μL of each phenol dilution were placed in duplicate wells of the assay plates. Artificial honey, sterile deionized water and catalase solution were included as negative controls. The plates were incubated at 37°C for 18 h.

The diameter of each zone of inhibition was measured using Vernier calipers. The mean diameter of the zone of inhibition around each well was squared, and a phenol standard curve was generated with phenol concentration against the mean squared diameter of the zone of inhibition. The activity of each honey sample was calculated using the standard curve. To account for the dilution and density of honey, this figure was multiplied by 4.69 (based on a mean honey density of 1.35 g/mL, as determined by Allen et al. (1991b), and the activity of the honey was then expressed as the equivalent phenol concentration (% w/v). Each honey sample was tested on at least three separate occasions, and the mean phenol equivalence was recorded.

### Assessment of antifungal activity

As there is no standardized method for assessing the antifungal activity of honey, this was done using the CLSI (formerly NCCLS) microdilution method with some modifications as described by Irish et al. (2006). This method, which has been developed to assess minimum inhibitory concentrations (MICs) of antibiotics, was used to assess the MIC of each honey against *Candida albicans* ATCC 10231 (Oxoid, Hampshire, UK). Briefly, honey samples were prepared by the addition of RPMI-1640 medium (Sigma-Aldrich, USA) to make 50% (w/v) stock solutions and incubated at 37°C with shaking at 200 rpm for 30 min to aid mixing. The diluted honey solutions were filter sterilized through 0.2 μm pore filters (Millipore) and further diluted with RPMI-1640 medium in 96-well U-bottomed microtitre plates to give final honey concentrations in 1% (w/v) increments from 10 to 50%. Artificial honey was included as an osmotic control.

*C. albicans* cultures were prepared by picking five colonies from an overnight yeast peptone dextrose agar plate and suspending them in 5 mL 0.85% saline. Transmittance of the culture was measured at 530 nm and adjusted to 0.8–0.88. Adjusted cultures were diluted 1:50 in sterile 0.85% saline, then further diluted 1:4 in RPMI-1640 medium, to achieve a working concentration of 5×10^3^ to 2.5×10^4^ cfu/mL. Twenty-five μL of the diluted culture was added to each well of the microtitre plate, resulting in a final inoculum of 0.5 to 2.5×10^3^ cfu/mL. Growth controls (no honey added) and sterility controls (RPMI-1640 medium only and honey solution only) were included in each plate. Following incubation at 35°C for 24 h, the MIC was recorded as the lowest concentration of honey that prevented growth, which was assessed visually. Each honey sample was tested in duplicate and the assays were repeated on at least three separate occasions, with the mean MIC recorded.

### Hydrogen peroxide assay

The concentration of H_2_O_2_ in honey samples was determined using a colorimetric assay that has previously been used to measure H_2_O_2_ in honey (Kwakman et al., 2010). Fifty percent (w/v) honey solutions were made by the addition of sterile deionized water to the honey samples. Samples were incubated at 37°C with shaking at 200 rpm for 30 min to aid mixing. The diluted honey solutions were filter sterilized through 0.2 μm pore filters (Millipore, USA) and further diluted to 25% (w/v) by mixing with either sterile deionized water or 5600 U/mL catalase solution (Sigma-Aldrich, USA). Aliquots of 40 μL of each honey sample were added to wells of a 96-well flat-bottomed microtiter plate in triplicate. H_2_O_2_ (Sigma-Aldrich, USA) standards ranging from 2.1 to 2200 μM were made by 2-fold serial dilutions and 40 μL of each standard was added to the plates. Sterile deionized water and catalase solution were also included as negative controls.

The reagent mixture consisting of 50 μg/mL of *O*-dianisidine (Sigma-Aldrich, USA) and 20 μ g/mL H_2_O_2_ type IV (Sigma-Aldrich, USA) in 10 mM phosphate buffer (pH 6.5), was freshly made from stock solutions of 1 mg/mL stock of *O*-dianisidine and 10 mg/mL of horseradish peroxidise type IV. One hundred and thirty-five μ L of this reagent mixture was added to wells of the microtiter plate containing the honey samples and H_2_O_2_ standards prepared as outlined above. Following incubation for 5 min at room temperature, reactions were stopped by the addition of 120 μL 6 M H_2_SO_4_. The color of the reaction was measured by absorbance at 560 nm using a Multiskan Ex plate reader (Thermo Scientific, USA), and H_2_O_2_ concentrations were calculated using a standard curve derived from the H_2_O_2_ standards. Each honey sample was tested in triplicate and assays were repeated on three separate occasions, giving a total of nine readings per honey sample.

### Statistical analysis

Statistical analysis of the data was performed using IBM SPSS Statistics 19 software. Differences between the activity of the honey samples and the artificial honey were evaluated using the independent samples *t*-test. This test was also used to compare the activity of the different honey types. Correlation analysis was done using Spearman's Rank Correlation with an online tool available at http://www.wessa.net (Wessa, 2011).

## Results

### Antibacterial activity of raw and processed honey samples

Four out of the 17 unprocessed honey samples (35%) had detectable antibacterial activity against *S. aureus* (Table 1). Activity in these samples was eliminated following the addition of catalase, and none exhibited detectable non-peroxide activity (data not shown). The four active honeys were red stringybark samples R1, R2, R3, and R6, and these had phenol equivalence values ranging from 12.0 to 21.2% (w/v). Following heat treatment and filtration, antibacterial activity was detected in only three red stringybark samples, and these were all significantly lower than the corresponding unprocessed honey samples. Activity was completely lost from sample R6, the red stringybark/canola blend (Table 1).

### Antifungal activity of raw and processed honey samples

All of the unprocessed honey samples had significantly higher antifungal activity than the artificial honey sample (*p* < 0.05; Table 1), however, this was also reduced to insignificant levels following catalase treatment (data not shown). The majority of the unprocessed honey samples had high MICs, corresponding to low antifungal activity, with 12 of the 17 honeys exhibiting MICs >30% (Table 1). Only two of the honeys (R2 and R3) had MICs <20% and three of the honeys (R1, R6, and Y3) had MICs <30%.

Most of the processed honey samples also had lower antifungal activity than the unprocessed honeys, and only nine samples remained more active than the artificial honey (Table 1). However, in red stringybark sample R1 the reverse was seen, and the processed sample was significantly more active than its unprocessed counterpart.

### Effect of processing on the production of hydrogen peroxide

H_2_O_2_ was measured in the honey samples before and after processing to determine how this was affected by heat processing. Eleven of the 17 unprocessed honey samples produced detectable H_2_O_2_ (Table 1). Four of the unprocessed red stringybark honey samples (R1, R2, R3, and R6) had among the highest H_2_O_2_ concentrations, which was consistent with the high antibacterial and antifungal activity seen in these samples However, H_2_O_2_ was also produced in unprocessed yellow box (Y3, Y4, and Y5), spotted gum (S1, S2, and S5) and canola honey (C1) samples that did not have any detectable antibacterial activity.

Correlation analysis using Spearman's Rank Correlation indicated that H_2_O_2_ production and antibacterial activity in the unprocessed honey samples was positively correlated (rho = 0.64; *p* = 0.005). However, this appeared to be driven by the samples with no H_2_O_2_ production, which also showed no antibacterial activity (samples R4, R5, Y1, Y2, S3, and S4). When these were removed from the analysis, correlation was lost (rho = 0.59, *p* = 0.056). In contrast, in the processed samples H_2_O_2_ production and antibacterial activity were strongly correlated across the entire dataset (rho = 0.77; *p* = 0.0002), and this remained (albeit reduced) when processed samples without H_2_O_2_ were excluded (rho = 0.88; *p* = 0.02). These results suggest one or more components are present in the unprocessed honey samples that modulate the inhibition of bacteria by H_2_O_2_, and these are sensitive to heat processing.

Antifungal activity was strongly correlated with H_2_O_2_ production in both the processed and the unprocessed samples (*p* < 0.005). However, this correlation was again lost when samples with no H_2_O_2_ production were removed from the analysis (rho = −0.53, *p* = 0.096; and rho = −0.71, *p* = 0.11 for unprocessed and processed samples, respectively).

Detectable H_2_O_2_ production remained in only six of the 17 honey samples following processing (Table 1). Red stringybark honeys R1, R2, R3, and R6 had the highest levels of H_2_O_2_, however, processing affected their H_2_O_2_ production differently, with a significant reduction seen in samples R3 and R6 but no significant change in samples R1 and R2. H_2_O_2_ was no longer produced by the majority of the processed yellow box and spotted gum samples, and it was undetectable in samples Y4, Y5, S5, and C1, which all had high H_2_O_2_ levels before processing yet had no detectable antibacterial activity. Overall, while it was apparent that heating the honeys negatively affected H_2_O_2_ production, the extent to which this happened varied in the different honey samples. Within the subset of samples where H_2_O_2_ was produced, there was no correlation between the amount produced before and after processing (rho = 0.46; *p* = 0.15).

## Discussion

There is increasing recognition of the value of medicinal honey, both as a high-value product that can be produced commercially in many parts of the world, including in rural and resource-poor settings, and as a potently active medicine that is effective against antibiotic-resistant pathogens. However, the parameters surrounding the reliable production of medicinally active honey remain poorly understood. Antimicrobial assays are usually performed on raw, unprocessed honey that is diluted and filtered to eliminate microorganisms prior to testing but is not heat-treated (Irish et al., 2011), but this may not be accurate if the honey must be heated subsequently to filter out particulate debris. In the current study, we assessed the antimicrobial properties of a number of independent samples of three common Australian honeys and investigated the effect of mild processing using heating and filtering methods that are routine for commercial honey production. In addition, as the current microbiological tests for antimicrobial activity are relatively labor-intensive, we analyzed whether floral source or H_2_O_2_ levels might be useful predictors of antimicrobial activity.

### Red stringybark: a useful floral source for medicinal honey?

Honey produced from native Australian flora has the potential for therapeutic use, firstly because a number of floral sources produce active honey (Lusby et al., 2005; Irish et al., 2011), and secondly because many Australian native forests occur in relatively remote areas that are likely to be free from pesticides and pollutants that could be introduced into the honey during production (Feás and Estevinho, 2011). Among the honeys selected for this study, some red stringybark samples displayed antibacterial activity at a potentially therapeutically useful level (Table 1; Molan, 1999). In a large-scale survey undertaken by Irish et al. (2011), different red stringybark samples had similar, relatively high antibacterial activities. However, in the current study there were large variations in activity among the different red stringybark samples. Similarly, although Irish et al. found Australian spotted gum honeys had antibacterial activity [median of 18.9% (w/v) phenol equivalence], none of the spotted gum samples in the current study showed activity. The current findings, and those from other studies (Allen et al., 1991a; Al-Jabri et al., 2003; Irish et al., 2011), indicate that while floral source is an important determinant of antimicrobial activity, it remains difficult to use this to predict the antimicrobial properties of a given honey sample. Therefore, while this work suggests that red stringybark could be a useful floral source for the production of medically active honey, the inconsistency among samples means individual samples still need to be screened for activity.

### Hydrogen peroxide production is not always sufficient for antimicrobial activity

As antimicrobial activity was reduced to insignificant levels when the honey samples were treated with catalase it was assumed that the production of H_2_O_2_ was responsible for most or all of the observed activity. In honey, glucose oxidase, which is secreted from the hypopharyngeal glands of bees, breaks down glucose to form gluconic acid and H_2_O_2_. Lack of free water and an acidic pH renders glucose oxidase inactive, but activity is restored when the honey is diluted with water, providing a slow, sustained release of H_2_O_2_, at sufficient levels to produce an antimicrobial effect but not high enough to damage mammalian tissues (Bang et al., 2003). In the current study, honey was diluted four-fold, which is optimal for H_2_O_2_ production from most honey types (Brudzynski et al., 2011).

Although there was a high level of correlation between the level of H_2_O_2_ produced by honey samples and their level of antibacterial and antifungal activity, which is consistent with other reports (White et al., 1963; Taormina et al., 2001; Brudzynski, 2006), this was lost once samples without any detectable H_2_O_2_ were excluded from the analysis; the only exception being the processed honey samples and their antibacterial activity. This suggests that H_2_O_2_ alone may not be sufficient for antimicrobial activity: honey samples with little or no H_2_O_2_ have a correspondingly low ability to inhibit bacteria and fungi, but if present, the level of H_2_O_2_ and the degree to which the honey is antimicrobial do not necessarily correlate. Indeed, some samples, such as canola honey C1 and yellowbox honey Y4 had particularly high H_2_O_2_ levels (754 and 645 μM, respectively) yet no antibacterial activity and very low antifungal activity, while sample R1 had 526 μM H_2_O_2_, but was among the most active of the honey samples.

Other studies have found that the level of H_2_O_2_ present in honey is more than 900-fold lower than expected based on the level of antimicrobial activity, and it has been suggested that there are one or more synergents present in honey that augment the action of H_2_O_2_ (Molan, 2006a; Kwakman et al., 2010; Brudzynski et al., 2011). It is possible that these synergents do not occur in samples where H_2_O_2_ was produced but little or no activity was seen. Alternatively, there may be other, as yet undefined compounds present in the inactive honey samples that interfere with the antimicrobial activity of H_2_O_2_. An interesting area of further study would be to compare the components present in honey samples with very different levels of antimicrobial activity but similar H_2_O_2_ levels (e.g., red stringybark sample R2 vs. canola honey C1), which may allow these possible synergents or agonists to be identified.

### Standard processing reduces the antimicrobial properties of honey but effect varies among samples

Processed honey samples had on average lower antifungal and antibacterial activity. Average antibacterial levels in the active samples (R1, R2, R3, and R6) dropped from 15.6 to 9.3% phenol equivalence. A significant reduction was seen in all but sample R1, and in sample R6, the red stringybark/canola blend, activity was lost altogether. Similarly, the average antifungal MIC changed from 31 to 33%, and for the canola honey C1, the MIC became significantly higher than for the artificial honey (Table 1; *p* < 0.05), indicating that this honey has less antifungal activity than an osmotically equivalent sugar solution. The change to antifungal activity following processing varied considerably among the different samples: only seven of the 17 samples dropped significantly in activity, nine were unchanged and in sample R1 the activity level significantly increased, with the MIC changing from 28.6 to 18 % (w/v) post-processing.

Heating above physiological temperatures is generally detrimental to enzymes, and a previous study on glucose oxidase in honey found that heating at 50°C for 20 min significantly reduced enzyme activity (Schepartz and Subers, 1964; White and Subers, 1964). Although most honey samples tested in the current study produced less H_2_O_2_ after heat treatment, with some dropping to zero, in others there was no significant difference before or after treatment, and overall there was no correlation between the level of H_2_O_2_ across the different honey samples pre- and post-heat treatment. Of interest is that while high levels of H_2_O_2_ were seen in some of the unprocessed samples that had no detectable antibacterial activity, only the active red stringybark samples (R1, R2, and R3) retained high (>500 μM) H_2_O_2_ levels post-processing. With only these three stably active samples, the current dataset is too small to derive robust conclusions. However, it is possible that the stability of H_2_O_2_ production is important in determining the activity of a honey sample, and a honey that loses the ability to produce H_2_O_2_ following standard heat processing could lose useful therapeutic activity, even if H_2_O_2_ levels prior to processing were high. Further investigation of this is warranted as a test to predict antibacterial activity based on H_2_O_2_ stability would be very helpful to the honey industry, and H_2_O_2_ levels alone appear to be a poor indicator of final activity levels.

The level of glucose oxidase in honey can vary depending on the health of the bees and the quality of their diet (Pernal and Currie, 2000; Alaux et al., 2010). However, the amount of H_2_O_2_ produced in a given honey sample is not determined by glucose oxidase alone, as honey can also contain catalase, peroxidases, and antioxidants such as gallic acid and caffeic acid that can degrade H_2_O_2_ or interfere with its ability to damage microbial cells (Weston, 2000; Al-Mamary et al., 2002; Sroka and Cisowski, 2003; Yao et al., 2003; Pyrzynska and Biesaga, 2009). In addition, it was recently reported that MGO directly modifies some proteinacious compounds in honey and if present this may also affect glucose oxidase activity (Majtan et al., 2012). The final level of H_2_O_2_ in a given honey sample therefore depends on various components, which can be present and active to varying degrees. Since any of these may be affected by honey processing, it is not unexpected that the different honey samples responded quite differently to heat treatment.

All commercial table honey is filtered to remove particulate debris, and heating up to 45°C is regularly used to increase the rate of filtration, but it is important to recognize that even relatively mild heat processing can reduce antimicrobial activity. Honey viscosity does not change appreciably above ~30°C (Matheson and Murray, 2011) and lower processing temperatures may be possible without a significant increase in inconvenience. Other studies have noted a reduction in enzymes, antioxidants and other phytonutrients following processing (Blasa et al., 2006; Turkmen et al., 2006; Ropa, 2010), and again this can vary considerably among samples. Minimal processing is therefore advisable for honey produced for medicinal purposes, and samples should be tested post-processing to ensure antimicrobial activity is not significantly reduced.

### Honey: a complex natural product

The complexity of natural products, including honey, makes them very difficult to standardize and this can affect their acceptance in clinical medicine. However, this complexity also has benefits. Unlike conventional antibiotics it appears to be difficult for microorganisms to become resistant to the effects of honey, probably due to the action of the various active components in honey on multiple microbial targets (Blair et al., 2009). An increasing interest in honey has led to recent studies that have begun to unravel how honey affects microbes at the cellular and molecular levels (Blair et al., 2009; Brudzynski et al., 2011; Kwakman et al., 2011; Kwakman and Zaat, 2012; Packer et al., 2012). New, advanced statistical methods for analyzing complex relationships may also help us to understand this complex process (Reshef et al., 2011). As well as developing a wider acceptance of selected honeys in conventional antimicrobial therapy, further studies could reveal lead compounds for the development of novel antimicrobials, which are urgently required.

## Conclusions

We conclude from this study that floral source and H_2_O_2_ levels, while important in determining the antimicrobial properties of honey, cannot be used to reliably predict whether a given honey sample will have antibacterial or antifungal activity. In general, processing with heat and filtration reduces H_2_O_2_-based activity but this varies in different honey samples. The most active honey samples produced high levels of H_2_O_2_ both before and after heating, suggesting H_2_O_2_ stability could be a useful indicator of antimicrobial activity, but further research with a greater number of samples is required to support this observation. The potentially detrimental effects of even mild heating should be taken into account when processing and testing honey destined for medicinal use.

### Conflict of interest statement

The authors declare that the research was conducted in the absence of any commercial or financial relationships that could be construed as a potential conflict of interest.
